# Magnetic Shape Memory Nanocomposites Assembled with High Speed High Pressure Torsion

**DOI:** 10.3390/nano14050405

**Published:** 2024-02-22

**Authors:** Carmela Gurau, Felicia Tolea, Nicanor Cimpoesu, Mihaela Sofronie, Alina Cantaragiu Ceoromila, Cristian Stefanescu, Gheorghe Gurau

**Affiliations:** 1 Department of Materials Engineering and Environment, Faculty of Engineering, “Dunarea de Jos” University of Galati, 47 Domneasca Street, RO-800008 Galati, Romania; carmela.gurau@ugal.ro (C.G.); cristian.stefanescu@ugal.ro (C.S.); 2 National Institute of Materials Physics, Atomistilor Street 405A, RO-077125 Bucharest-Magurele, Romania; felicia.tolea@infim.ro (F.T.); mihsof@infim.ro (M.S.); 3 Department of Materials Science, Gheorghe Asachi Technical University of Iasi, Bd. Dimitrie Mangeron, nr. 67, RO-700050 Iasi, Romania; nicanor.cimpoesu@tuiasi.ro; 4 Applied Sciences Department, Cross-Border Faculty, “Dunarea de Jos” University of Galati, 47 Domneasca Street, RO-800008 Galati, Romania; alina.cantaragiu@ugal.ro

**Keywords:** severe plastic deformation, HSHPT, magnetic shape memory composite

## Abstract

When a severe plastic deformation (SPD) process is performed at high temperatures, it becomes more versatile. Designed originally for the bulk nanoconstruction of hard-to-deform alloys, high-speed high-pressure torsion (HSHPT) is an SPD method used in this research for assembling multiple layers of shape memory nanocomposites. Three hard-to-deform magnetic alloys in the cast state were used. Soft magnetic shape memory alloys, NiFeGa and FePdMn, and a potentially hard magnetic alloy, CoZr, were assembled in various composites. Both grain refinement and strong layer bonding were achieved in ZrCo/FePdMn and ZrCo/NiFeGa composites in seconds. The very short SPD time is specific to HSHPT because of the intense friction that occurs under high pressures, which generates huge amounts of heat. After SPD, the temperature rises in bulk material like a pulse, being dissipated mostly through heat conduction. The SPD parameters were carefully controlled with an advanced automation system using a programmable logic controller. Nevertheless, the major drawbacks of high-pressure torsion were overcome, and large SPD discs were obtained. Various investigation techniques (optical microscopy, scanning electron microscopy, energy dispersive spectroscopy and atomic force microscopy) show well-defined interfaces as well as a fine and ultrafine structure.

## 1. Introduction

Severe plastic deformation (SPD) techniques overcome the challenges of developing multimetal composites with new combinations of properties and therefore wider functionality [1,2,3,4]. SPD particularly allows for the generation of multilayered metallic composites with nanostructured components possessing advanced properties [5,6,7]. Remarkable results can also be obtained by using a hybrid SPD method, namely high-speed high-pressure torsion (HSHPT) [8]. HSHPT is based on high-pressure torsion (HPT), ignoring the slippage of the tool–sample surface. This key observation is the main difference between HPT and HSHPT. In HSHPT, the extensive friction at high pressures is the source of the heat pulse generated inside the sample in seconds. Recrystallization in this hot SPD is very limited [9]. The bulk material flows very fast on the cold surface of the tools, with the flow occurring in adiabatic conditions. The heat is dissipated via conduction, with the heat transfer being accelerated by very thin-walled samples. Also, the relatively large diameter of the discs (up to 40 mm) compared with traditional HPT discs (around 20 mm in diameter) favors heat transfer [9,10,11,12]. HSHPT has demonstrated this unique capability of bonding in solid-state dissimilar alloys and hard-to-deform–hard-to-weld (NiTi/NiTi, NiTi/NiFeGa) materials [8,9]. The tool–sample surface is protected to avoid adherence, but interfaces between layers are activated by friction under high pressures, leading to new, strong atomic bonds. HSHPT technology enables the joining of layers via thermocompression. On the micro scale, very effective and clear interfaces may be observed.

The main objective of our study was to evaluate the possibility of producing multilayered metallic composites between dissimilar layers of two different soft ferromagnetic shape memory alloys (FSMAs) and the Co87Zr13 magnetic alloy. FSMAs are known for their shape memory effect combined with a large magnetic field-induced strain as materials for rapid magnetic actuators [13]. The Fe-Pd (30 at.%Pd)-based FSMA has been highlighted because of its high ductility, corrosion resistance, low brittleness, biocompatibility and moderate magnetic field-induced strain at room temperature. A thermo-elastic and reversible martensitic transformation from an austenite phase with a face-centered cubic (f.c.c.) structure and a martensite phase with a face-centered tetragonal (f.c.t.) structure is initiated via cooling near room temperature. In the Fe67Pd30Mn3 FSMA, a small addition of Mn increases the characteristic transformation temperatures, keeping the ferromagnetic behavior with implications for potential applications [13,14,15,16]. The other is Ni55Fe20Ga25, which has been linked with a reversible martensitic transformation between the B2 austenite and tetragonal structure [17,18]. The bonding of topologically different layers was produced through HSHPT. However, our previous works were devoted to the detailed investigation of the structural and thermomagnetic properties associated with the reversible martensitic transformation of NiFeGaAl and FeMnPd soft magnetic SMAs after HSHPT [19]. Furthermore, the characteristic martensitic transformation temperatures and the magnetic ordering temperatures, depending on the applied true strain, were investigated. A study on the morphology and magnetic properties of the Co87Zr13 magnetic alloy, which was severely plastic-deformed using the HSHPT technique, was previously published [20]. The study reported improvements in the structural, magnetic and mechanical properties after HSHPT. New properties could be created in composites which combine these severely plastic-deformed alloys in order to achieve wider functionality [21,22,23,24].

This research is intended to fabricate multilayered composites consisting of a Co87Zr13 magnetic alloy and two types of soft MSMAs metallurgically joined with HSHPT. This study investigated the mixing of layers depending on the alloy composition and the degree of deformation. The evolution of the structure and mechanical properties in each layer of the MSMC and at the boundaries between soft and potentially hard magnetic multilayers was examined.

## 2. Materials and Methods

Bulk samples of Co87Zr13, Ni52Fe20Ga23Al5 and FePd30Mn3 were elaborated via arc melting in protective atmosphere (Ar) starting from high-purity elements (99.99%) produced by Alfa Aesar, Karlsruhe, Germany. For higher homogeneity, the alloys were remelted five times.

Cast bulk alloys were subjected to HSHPT to obtain SPD-ed disks. The thin disks (under 0.1 mm thick and with a 22 mm diameter) were finally assembled in Co87Zr13/FePd28Mn3/Co87Zr13 and Co87Zr13/Ni52Fe20Ga23Al5/Co87Zr13 magnetic shape memory composites using the same technique. The HSHPT equipment is powered with a 15 kW AC motor directly coupled with the upper-HSHPT punch. The upper anvil rotation speed varies between 10^2^ and 2 × 10^3^ rpm and is controlled with the EATON SVX024A1-4A1B1 frequency converter (Dublin, Ireland). The lower punch is hydraulically driven, and the maximum force for the Co87Zr13/FePd30Mn3/Co87Zr13 composite and Co87Zr13/Ni52Fe20Ga23Al5/Co87Zr13 composite is 17.403 kN and 15.520 kN, respectively. The displacement of the lower anvil is controlled with the Li500 Q25LMO LiU5X3 H151 sensor from TURK (Grünhain-Beierfeld, Germany). The pressure (0.483 GPa for Co87Zr13/FePd30Mn3/Co87Zr13 and 0.454 GPa for Co87Zr13/Ni52Fe20Ga23Al5/Co87Zr13) was monitored with the Hottinger Spider 8 system and Catman 5.0 software, also from Hottinger, Darmstadt, Germany. The stability of HSHPT parameters in multiple experiments is based on integrated signals in a smart automation system using PLC XC 200 from EATON and a homemade graphical interface, XSOFT-CODESYS V2.3.9 SP4. The temperature was monitored with a CT LASER type OPTC T2 MHCF (OPTRIS) laser sensor (Berlin Germany). The temperature was under the detection limit (385 °C) during the HSHPT process of fabrication of shape memory magnetic composites.

Optical microscopy was carried out on an OLIMPUSBX51 microscope (Tokyo, Japan) The microscope is equipped with a camera controlled with QuickPHOTO MICRO 2.3 software.

Scanning electron microscopy (SEM) was used to investigate the severe plastic deformation samples using a secondary electron (SE) detector and a cathode power supply of 30 kV in a high-vacuum chamber. The equipment is a Vegatescan LMH type II (TESCAN, Brno-Kohoutovice, Czech Republic) device with VegaTC 3.5.19.0 software.

Chemical composition determination was conducted using an EDS (energy dispersive spectroscopy) retractable detector from Bruker (Ettlingen, Germany), specifically the X-Flash 6–10 model from Germany. For determination, automatic (precise mode), line and mapping modes were employed, and for analysis, Esprit 2.2.0.3007 software (Berlin, Germany) was utilized. To enhance the quality of results, Quanta 200 from Thermo-Fischer Scientific SEM (Waltham, MA, USA) was also employed.

For SEM imaging analysis, gold alloy sputter-coating was required (Sputter Coater, SPI Supplies, West Chester, PA, USA). The high-resolution images were captured through the secondary electron signal generated by a beam accelerated at 15 kV voltage in vacuum conditions at 60 Pa pressure. In order to provide the microchemical composition, SEM microstructural analysis was coupled with EDS.

Atomic force microscopy (AFM), type EasyScan II from Nanosurf, Switzerland, was used to scan the profile of the samples’ surfaces. Scans were performed at different magnifications using a contact Si tip type ppp-CONTR 10.

The microhardness HV0.1 test was performed under a 0.980665 N load for 15 s at room temperature considering the mean value of three measurements. Microhardness equipment PMT3 (Neftekamsk, Russia) with an L3CMOS14000KPA 14MP digital camera (Oxfordshire, UK) and ToupView 2.0 software was employed for Vickers measurements.

## 3. Results and Discussion

### 3.1. Severe Plastic Deformation with High-Speed High-Pressure Torsion

Magnetic shape memory nanocomposites were produced in two steps. First, as-cast Co87Zr13, Ni52Fe20Ga23Al5 and FePd30Mn3 were severe plastic deformed in thin disks. Previously, we have presented extensively the severe plastic deformation of those hard-to-deform magnetic shape memory alloys [19,20,21]. The aim of the present study was to investigate in detail the assembling process of various magnetic composites.

**Figure 1:**
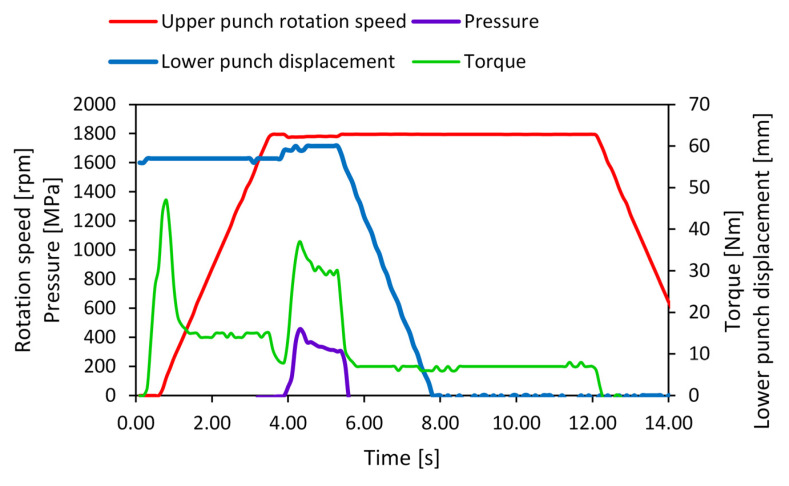
Variation in HSHPT parameters versus time.

For example, to obtain the Co87Zr13/Ni52Fe20Ga23Al5/Co87Zr13 composite, three disks were made to overlap alternatively. This sandwich stack was pressed in the HSHPT machine prior to SPD. The lower punch went up to 57 mm from the rest position (Figure 1 dot line plot). After that, the initial force was removed, and the upper punch started rotating at 1795 rpm. After 4 s, full pressure was applied (0.454 GPa), and the solid-state bonding began. As we can see in Figure 1, the pressure decreased to 0.303 GPa at the end of the process. The entire process takes two seconds. The rotation speed plot shows a slightly disturbed plateau (1781 rpm) during SPD because a large amount of power was absorbed for the disk welding. The torque was measured on a motor shaft and presents two peaks. The first peak is related to the break in adhesion between the disks, which start rotating in contact with the upper anvil. The second peak appears when the bonding process begins. The torque profile is very similar to the pressure profile.

### 3.2. Optical Microscopy

**Figure 2:**
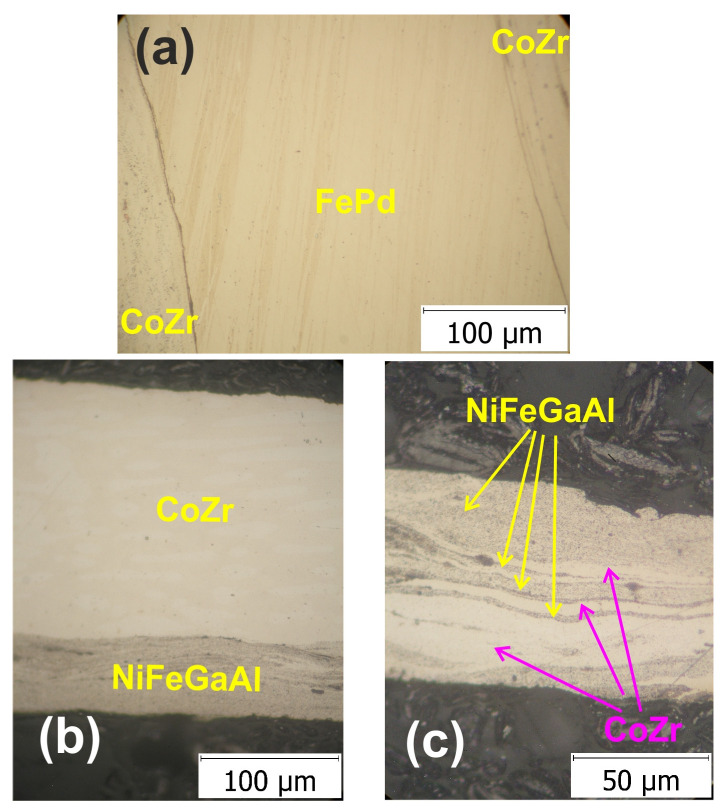
Optical micrographs of (**a**) the three-layered CoZr/FePdMn/CoZr composite disk, (**b**) CoZr/NiFeGaAl bilayered composite disk and (**c**) CoZr/NiFeGaAlCoZr13 multilayered composite disk.

One of the sandwich blanks was prepared by overlapping the three disks: one disk of the FePd30Mn3 soft magnetic alloy between two disks of the Co87Zr13 magnetic alloy. Prior to producing the composite via HSHPT severe plastic deformation, all three disks were also deformed with the same deformation degree (ε = 0.46) on the same machine from cast raw materials. The cumulative degree of deformation of the composite was 3.98. The OM of the resulting three-layer MSMC is illustrated in Figure 2a. The structure was studied in the cross-section around the middle of the disk. The micrograph of the three-layered Co87Zr13/FePd30Mn3/Co87Zr13 alloy composite reveals a refined microstructure proving a typical severe plastic deformation structure. The grain boundaries could not be identified as the size of these microstructural features was outside the resolution range of the optical microscope. However, only straight lines (oriented approximately 60° to the interface) could be observed across the thickness of the soft magnetic alloy layer in the middle of the composite. On the surface of the soft magnetic SMA, a finer structure can be noticed, comprising randomly arranged dashed flow lines. The CoZr magnetic layer exhibits a fine structure with no visible grain boundaries, only discontinued flow lines. The bonding interface between the layers is narrow. In addition, a smooth and continuous interface between layers with an almost linear shape was observed. The second part of this study involved the production of Co87Zr13/Ni52Fe20Ga23Al5/Co87Zr13 multilayered magnetic composites. These MSMCs were fabricated by alternatively overlapping two and three disks of the Ni52Fe20Ga23Al5 soft alloy and Co87Zr13 magnetic alloy. The disks were also deformed from as-cast materials. The optical image of the two layered composite specimens (Figure 2b) has two distinctive layers much thinner than in the initial sandwich blank.

The degree of deformation integrated in the Co87Zr13/Ni52Fe20Ga23Al5 bilayered composite was 2.46. The microstructure of the NiFeGa layer illustrates the dual phase consisting of a very fine martensitic matrix and elongated γ phase. There is a clear demarcation between the two layers, with a slightly sinusoidal appearance. In Figure 2c, the disk of the Ni52Fe20Ga23Al5 soft alloy is shown alongside two disks of the Co87Zr13 magnetic alloy. The cumulative degree of deformation was similar to that of the three-layered Co87Zr13/FePd30Mn3/Co87Zr13 composite, 3.97. The micrograph shows a refined microstructure in keeping with the expected features after severe plastic deformation produced via HSHPT [13]. The structure obtained in the other composites under study is quite different from the layout of the CoZr/FePdMn/CoZr alloy composite (Figure 2c). The layers changed not only in thickness; they also mixed and formed a multilayered configuration. The structure is a result of the fragmentation of layers owing to the high deformation degree applied using HSHPT. The thinner layers are mixed in a sinuous shape and in some of the vortex area. It was found that there is an intercalation of the layers that is better highlighted with EDS analysis. This is associated with the increase in the cumulative degree of deformation in the case of composites fabricated from dissimilar alloys, which is characteristic of HSHPT.

### 3.3. SEM Investigation of MSMC Composites

**Figure 3:**
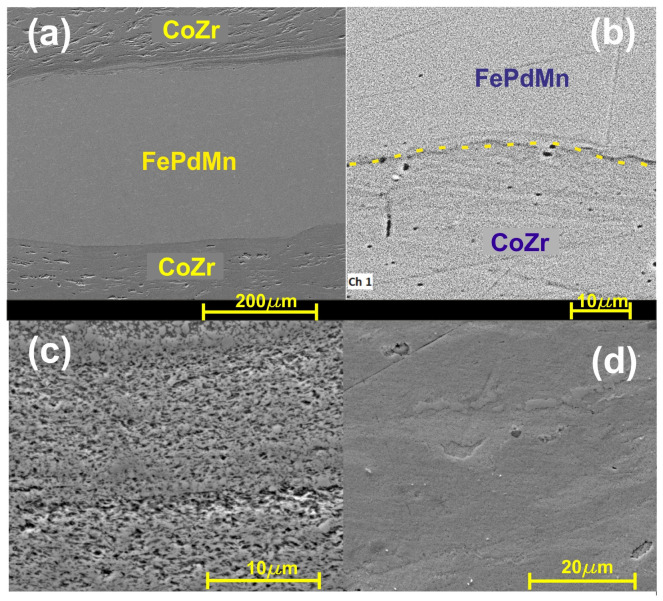
Cross-sectional secondary electron image of (**a**) CoZr/FePdMn/CoZr trilayered composite, (**b**) interface of FePdMn/CoZr (yellow dashed line), (**c**) focus on the CoZr layer and (**d**) on the FePdMn layer.

It is widely known that SPD processing involves significant refinement of the microstructure. The HSHPT-SPD process of the CoZr/FePdMn MSMC, exhibiting a 3.98 logarithmic degree of deformation, illustrates the morphology of the highly deformed microstructure observed under the scanning electron microscope (Figure 3).

The cross-sectional secondary electron image of the composite highlights a very fine structure of trilayered composite with two distinct interfacial regions. The aspect of the bonding area is smooth and continuous. In the upper part of the micrograph, the interface shows a multilayered area composed of four extremely thin alternative layers. This feature of the structure formed after HSHPT is caused by the fragmentation and slipping of neighboring layers, like in the case of the CoZr/NiFeGaAl MSMC. Both composites exhibit almost the same logarithmic degree of high deformation. Figure 3b illustrates a uniform and slightly curved interfacial zone. In this case, a few precipitates can be observed along the bonding area. The fine structure is visible in all layers; however, the FePdMn layer is denser, finer and more homogeneous.

**Figure 4:**
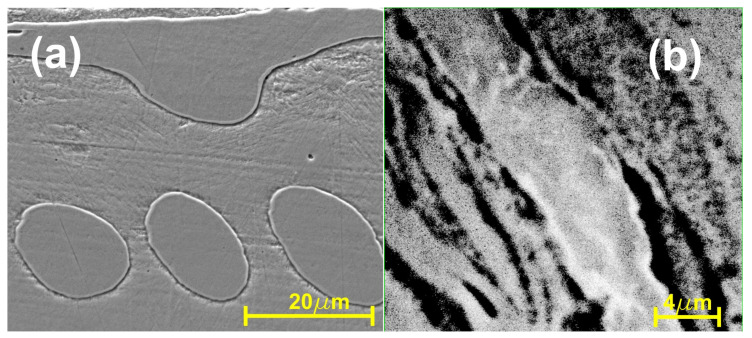
Cross-sectional secondary electron image of CoZr/NiFeGaAl: (**a**) bilayered composite focused on the NiFeGaAl layer, (**b**) multilayered composite.

In the bilayered composite specimen that was subjected to a 2.01 logarithmic degree of deformation (Figure 4a), the cross-section of the NiFeGaAl layer manifests γ precipitate, which has a round or elongated shape inside the fine martensite matrix.

A pronounced grain refinement could be observed in the CoZr/NiFeGaAl MSMC subjected to a logarithmic deformation degree level of 3.97 as compared with the bilayered specimen (Figure 4b). The HSHPT processing involved grain refinement via shearing and fragmenting of multilayered composites. The micrograph shows only curved fibering and a few vortex areas. The layers of the two alloys could not be properly identified, in contrast. However, as can be seen from the EDS study, the layers of CoZr were interpenetrated with NiFeGaAl adjacent bands.

**Figure 5:**
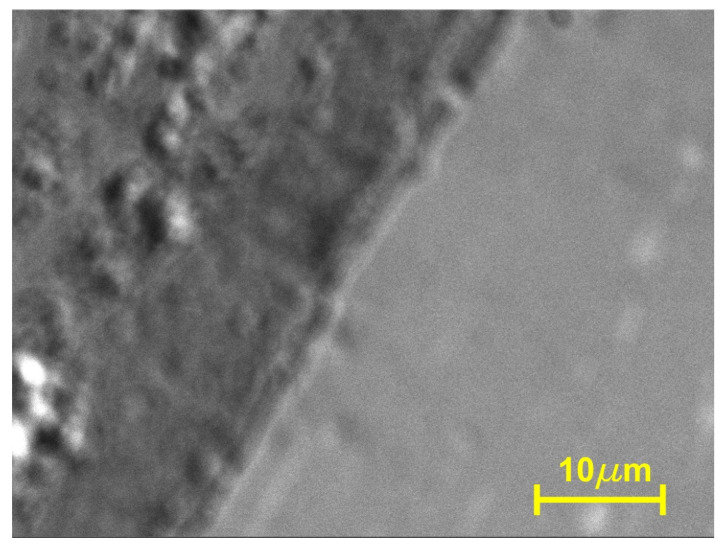
SEM micrographs illustrating the interfacial zone of the composite CoZr/FePdMn: general aspect.

The micrographs of the CoZr/FePdMn MSMC show a distinct interfacial region (Figure 5). The continuity of the bonding is noticeable. Both layers demonstrate very fine structures possessing a narrow, straight and continuous boundary between them. The interface has a restricted thickness determined only by cobalt and zirconium diffusion at a short distance, confirmed via EDS line scan analyses. This type of narrow interface is specific to HSHPT processing and lasts about 2 s. The very short time of deformation leads to a very low diffusion rate. HSHPT severe plastic deformation combines high pressure (about 1 GPa) with shear straining caused by a high rotation speed, which increases the temperature and breaks down the oxide layers found on the surface between CoZr and SMA disks. The good contact between the sandwich disks, enhanced by the high pressure, the increased temperature due to friction and the extremely short time of deformation, lead to the consolidation of the composite and to strong bonding of hard and soft magnetic layers.

### 3.4. SEM-EDS Analysis

**Figure 6:**
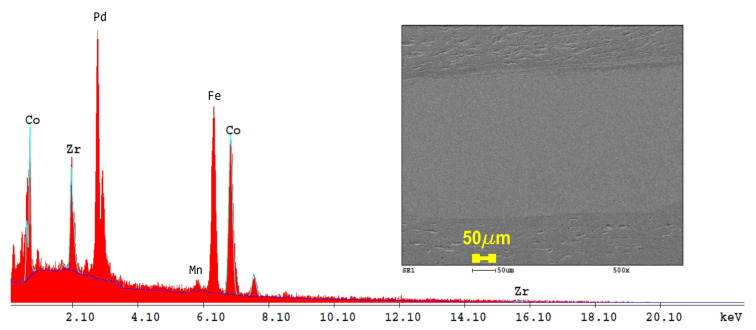
Results of SEM analysis of a Co87Zr13/FePd30Mn3/Co87Zr13 composite: SEM micrograph of a cross-section and elemental spectrum of scanned area.

Figure 6 shows representative SEM-EDS images on the surface of the three-layered Co87Zr13/FePd30Mn3/Co87Zr13 alloy composite. During severe plastic deformation conducted with HSHPT, the initial disks in close contact became a fully consolidated composite. The high rotation speed of 1796 rot/min and concomitantly applied high pressure (0.7 GPa) established the conditions for metallurgical bonding of these dissimilar magnetic alloys. It is noticeable in Figure 6 that the composite has three well-outlined layers. The grain refinement process, after SPD, is fairly visible. The soft magnetic alloy possesses the densest structure, being in accordance with the measured results for microhardness analyses (Section 3.6). However, CoZr also shows short, sinuous flowlines. These dissimilar layers became interconnected through neighboring atoms without a transition region, distinguishable via SEM analysis. It is obvious that there is a continuous boundary between the FePdMn and CoZr layers. Only a very thin CoZr layer penetrated the FePdMn layer, as can be seen in the upper part of Figure 6. The elemental spectrum of the layers after HSHPT consolidation is also indicated.

**Figure 7:**
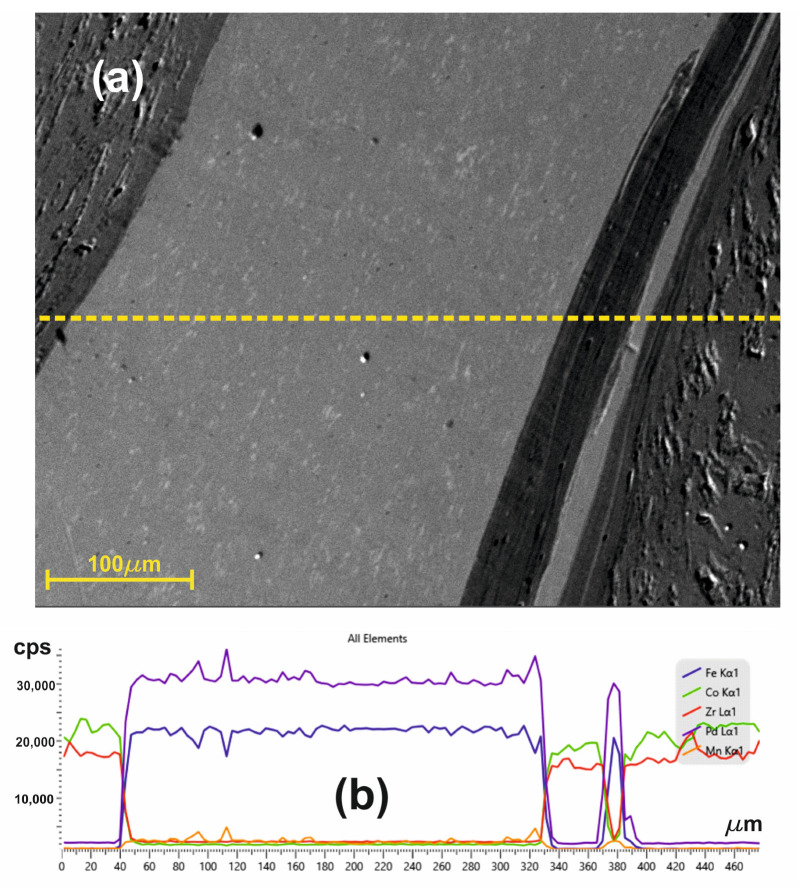
(**a**) SEM image at a higher magnification of CoZr/FePdMn/CoZr composite in an area with five layers and (**b**) EDS line scan across these adjacent layers.

To understand the influence of HSHPT on the structure of the CoZr/FePdMn composite that was subjected to a 3.98 logarithmic degree of deformation, an SEM-EDS investigation was performed in an area with three layers clearly noticed in the top of the micrograph shown in Figure 7.

The SEM image, recorded at a higher magnification, highlights through strong color contrast the presence of a very thin layer of CoZr which has penetrated the FePdMn layer. Only in this area does the MSMC comprise five distinct layers. The succession of layers is better emphasized with the EDS line scan performed along a line crossing the five neighboring layers (Figure 7 yellow dashed line). Throughout the whole zone comprising these five adjacent layers, alternative, rich portions can be identified that contain the chemical elements from the composition of CoZr or FePdMn alloys that form the MSMC. It can be observed that none of the chemical elements of one alloy were found in the layer corresponding to the other alloy. The atomic migration of the constitutive chemical elements took place only over very short distances (≤5 µm) in the bonding area of the layers. In the interfacial zones, evidence could be found for diffusion occurrence, which favored an excellent metallurgical joining between the studied magnetic alloys. The bonding interfaces were narrow and continuous. An abrupt increase in Fe and Pd content could be noticed at interfaces of the soft magnetic SMA. However, the mobility of Co and Zr near interfaces is supported by the occurrence of a slight slope.

**Figure 8:**
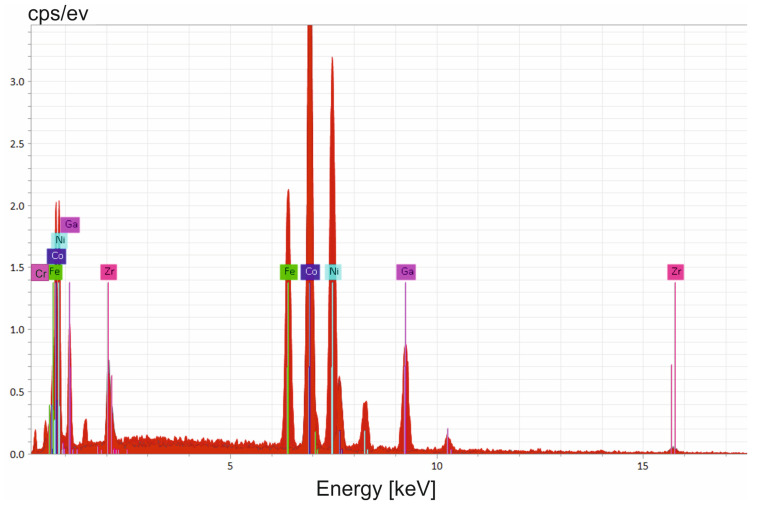
EDS patterns of the scanned area from the Co87Zr13/Ni52Fe20Ga23Al5/Co87Zr13 composite.

The MSMC was fabricated by encasing a Ni52Fe20Ga23Al5 soft magnetic alloy between two Co87Zr13 magnetic alloys, and the process involved a 3.97-degree deformation, matching the deformation of the CoZr/FePdMn/CoZr composite. An EDS analysis was used to study the structure of the sample cross-section of the Co87Zr13/Ni52Fe20Ga23Al5/Co87Zr13 composite disk (Figure 8). In the area comprising the composite multilayers, only the spectral lines of the chemical elements that constitute the magnetic alloys can be distinguished.

**Figure 9:**
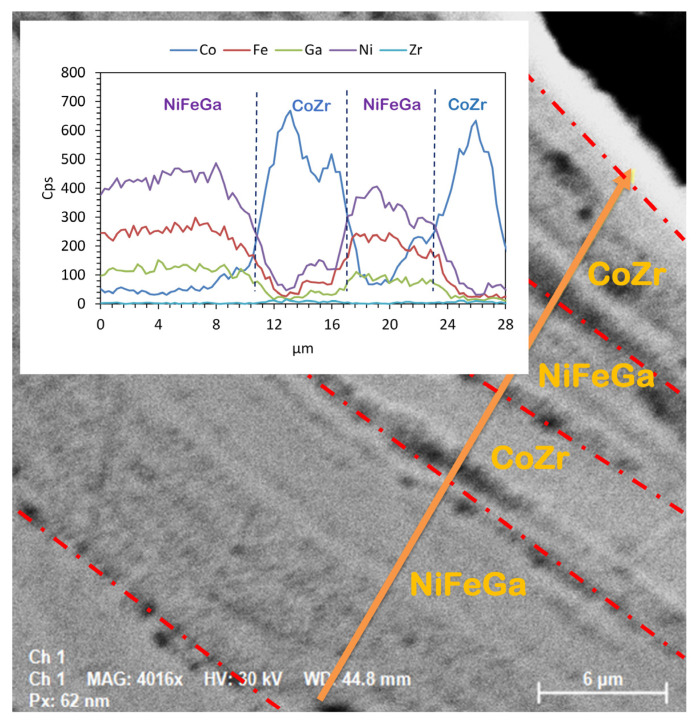
SEM image of Co87Zr13/Ni52Fe20Ga23Al5/Co87Zr13 MSMC and inside frame of the EDS line scan across the adjacent layers (red dashed lines).

The Co87Zr13/Ni52Fe20Ga23Al5/Co87Zr13 multilayered magnetic composite subjected to HSHPT reveals a noticeable grain refinement process (Figure 9). The SEM microstructure of the composite processed using HSHPT shows typical ultrafine features resulting from severe plastic deformation. Although the initial composite comprised only three severely deformed disks, after HSHPT the resulting composite presented a multilayered structure as can be noticed in Figure 9. The joint between the sheets is narrow and discontinuous and outlined by some precipitates. To investigate the distribution of elements in the multilayered CoZr/NiFeGaAl, an EDS line scan was performed along a surface comprising four neighboring layers. In all of the studied regions, Fe, Ni, Ga and Co atoms can be identified. However, the Zr content is concentrated only in areas corresponding to CoZr layers. Fe, Ni and Ga are distributed rather uniformly throughout the soft magnetic layers and decreased monotonically inside the potential CoZr magnetic layers. Co atoms are rather uniformly distributed in the NiFeGa layers with peaks indicating significantly increased content within the CoZr magnetic layers. The element distribution profiles of Co and Zr and Fe, Ni and Ga show a negative correlation. Nevertheless, traces of compounds of the soft magnetic alloy were highlighted along CoZr magnetic layers.

**Figure 10:**
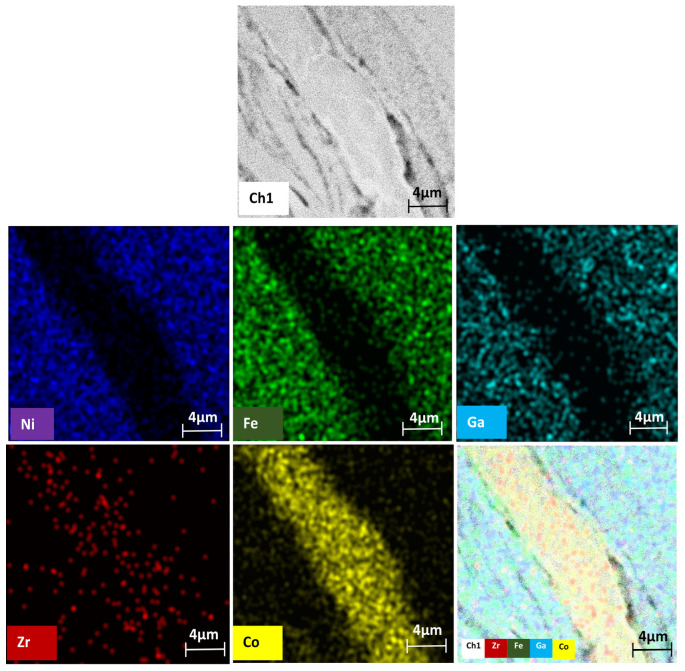
EDX mapping of the chemical element distribution of the CoZr/NiFeGa multilayered composite.

As suggested from the arrangement of the chemical element distribution on the EDS map (Figure 10), the composite layers were metallurgically well bonded with little evidence of interfacial reactions in the connection zones. In the middle of the SEM surface, only Co and Zr were observed as being rather uniformly distributed over the layer area. In the adjacent layers of the surface of the composite, Fe, Ni and Ga, the constituents of the soft magnetic layer, were noticeable. Additionally, Zr, which migrated long distances inside adjacent soft magnetic layers, could be observed. Across the layer boundaries, narrow strips of Fe-rich chemical compounds could be identified. Inside the NiFeGa layer, discontinuously arranged narrow and meandering Ni-rich compounds were found. In addition, Fe-rich curved zones could be identified.

An interesting aspect revealed by the SEM-EDS investigation was the distinct manner of the two magnetic shape memory alloys during the buildup of the composite with CoZr. Although the HSHPT deformation was promoted on three packed disks, the resulting FePdMn/CoZr composite consisted of three definite layers, while the NiFeGa/CoZr composite was made up of multiple sinuous layers. The different persisting structures, resulting from the same severe plastic deformation process, are probably due to the distinct flow mechanisms produced through severe torsion rotation under high pressure. The harsh environment led to the deformation of multilayered mixed structures as a result of the fragmentation of the NiFeGa alloy into thin corrugated layers joined between CoZr magnetic layers. This finding is in good agreement with early work on steel composites with various combinations of metals [1]. In the case of the FePdMn/CoZr composite, the bonding area observed was smooth, with only one narrow CoZr layer diffused inside the soft magnetic middle layer near the joining area. The manner of welding and the mechanism of consolidation of layers was conducted under severe plastic deformation conditions and with differing types of materials.

### 3.5. AFM Study of CoZr/NiFeGaAl Multilayered Composite

**Figure 11:**
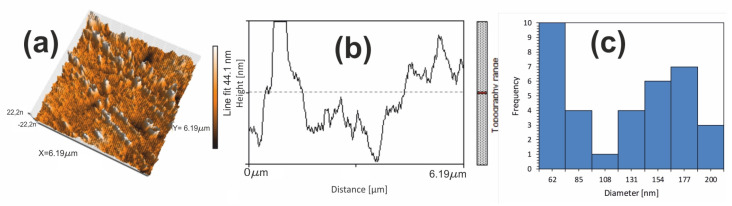
(**a**) CoZr/NiFeGaAl multilayered composite 3D image scanned with atomic force microscopy (AFM), (**b**) height section of the line in (**a**) showing the topographical changes, (**c**) grain size distribution histogram for the magnetic shape memory multilayered composite.

The atomic force microscopy morphology investigation of the 6.19 µm × 6.19 µm region from the CoZr/NiFeGaAl multilayered composite is presented in Figure 11a. The difference in surface roughness suggests an alternative layer structure of the MSMC.

This observation is consistent with the finding from the SEM-EDX investigation. The height cross-section profile of the two dissimilar alloys was 44.1 nm (Figure 11b). The mean grain size was calculated with the midpoints of the histogram. As revealed with a statistical grain size analysis (Figure 11c), the grain size distribution is in the range of 43–191 nm. The aspect of the histogram of the statistical grain distribution can be explained by two Gaussian frequency curves belonging to each component alloy of the MSMC. At the same degree of deformation, the alloy with the lower hardness showed a finer structure. The average grain size below 100 nm was 43%. The composite demonstrated an ultrafine grained structure, with grain sizes under 200 nm.

### 3.6. Mechanical Properties of MSMCs

The Vickers microhardness was recorded along with the thickness of the studied MSMCs. The microhardness test after HSHPT on the dissimilar layers revealed a value of 270 HV for the FePdMn and a value of 555 HV for the CoZr layers. Both CoZr outer layers acted as reinforced zones of the composite, being harder than the inner FePdMn layer. The hardness of the CoZr magnetic alloy was 2.05 times larger than the magnetic shape memory alloy. The interface area showed a 660 HV higher value of microhardness than even the CoZr layers. The elemental diffusion across interfaces observed in the EDX study can be involved in the occurrence of the limited, harder region. This finding was reported only for the interface with a very thin layer of CoZr, which penetrated the FePdMn layer. The microhardness results of the CoZr/NiFeGaAl bilayered and multilayered composites processed with HSHPT at different logarithmic degrees of deformation are in good agreement with the refined microstructure. The hardness of both layers was significantly higher, with an increase in the level of the degree of deformation from 2.46 to 3.97. Microhardness tests showed reinforcement of NiFeGaAl layers from 351 HV to 398 HV (1.13 times). A change in the microhardness of CoZr layers took place from 518 HV to 612 HV (1.18 times). The difference in the microhardness value is associated with the decrease in grain size at the higher imposed strain. The hardness of CoZr magnetic layers was 1.53 times that of NiFeGaAl layers. As compared with the CoZr/FePdMn composite at the same imposed strain, the difference in the hardness values between layers is lower. The softer layer is more ductile, forming a three-layered Co87Zr13/FePd30Mn3/Co87Zr13 composite disk. In the case of the composite with harder layers of the NiFeGaAl magnetic shape memory alloy, interpenetration of dissimilar layers into the multilayered composite takes place.

## 4. Conclusions

The primary findings on the magnetic shape memory composites presented in this study can be summarized in the following points:

1. The HSHPT severe plastic deformation technique was successfully used to fabricate novel multilayered magnetic shape memory composites. The manufactured multilayered metallic composites are composed of a Co87Zr13 magnetic alloy and one of the two soft ferromagnetic shape memory alloys, FePd30Mn3 or Ni55Fe20Ga25.
2. The initial cast magnetic alloys were subjected to HSHPT with the same deformation degree (ε = 0.46) in disks. The second step was fabricating the MSMCs from the initial disks with 2.46 and 3.97–3.98 logarithmic deformation degrees. The resulting composites demonstrate strong metallurgical joining of all dissimilar magnetic layers.
3. The difference between the hardness values of layers was involved in the consolidation process of the composites. The softer layers, which are more ductile, formed distinct layers. The harder layers determined fragmentation and interpenetration of layers into multilayered composites.

Finally, we stress that the unique combinations of potentially hard magnetic layers with soft magnetic shape memory layers demonstrates the potential for new hybrid ultrafine grained composites with enhanced proprieties of high interest for applications.

## Data Availability

The raw data supporting the conclusions of this article will be made available by the authors on request.
